# Bis{2,2′-[methyl­aza­nediylbis(methyl­ene)]bis­(4,6-dimethyl­phenolato)-κ^3^ *O*,*N*,*O*′}titanium(IV) toluene sesquisolvate

**DOI:** 10.1107/S1600536813007022

**Published:** 2013-03-20

**Authors:** Yongseog Chung, Youngjo Kim

**Affiliations:** a Department of Chemistry, Chungbuk National University, Cheongju, Chungbuk 361-763, Republic of Korea

## Abstract

The title compound, [Ti(C_19_H_23_NO_2_)_2_]·1.5C_7_H_8_, crystallizes with one titanium complex mol­ecule per asymmetric unit together with one and a half toluene mol­ecules. The Ti^IV^ atom is coordinated by two fully deprotonated *O*,*N*,*O*′-tridentate phen­oxy­amine ligands in a distorted octa­hedral environment. Within this arrangement the O atoms occupy the equatorial sites and the N atoms the axial sites. One of the toluene mol­ecules is disordered over two sets of sites in a 0.628 (18):0.372 (18) ratio.

## Related literature

For other compounds of titanium with tri- and tetra­dentate ligands, see: Mun *et al.* (2010 ▶); Chmura *et al.* (2006 ▶); Hong *et al.* (2008 ▶); Kim *et al.* (2009 ▶, 2011 ▶, 2012 ▶); Lee *et al.* (2007 ▶, 2008 ▶).
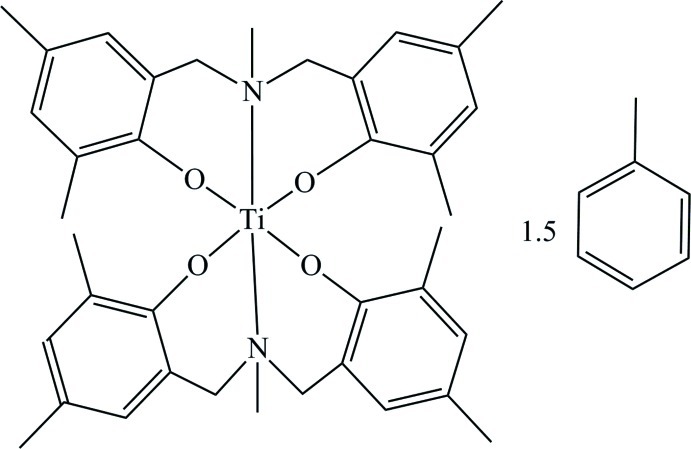


## Experimental

### 

#### Crystal data

[Ti(C_19_H_23_NO_2_)_2_]·1.5C_7_H_8_
*M* *_r_* = 780.34
Triclinic, 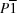

*a* = 12.053 (2) Å
*b* = 13.374 (3) Å
*c* = 14.511 (3) Å
α = 102.02 (3)°
β = 112.07 (3)°
γ = 93.04 (3)°
*V* = 2097.8 (7) Å^3^
*Z* = 2
Mo *K*α radiation
μ = 0.25 mm^−1^
*T* = 173 K
0.50 × 0.25 × 0.14 mm

#### Data collection

Bruker SMART CCD area-detector diffractometer
Absorption correction: multi-scan (*SADABS*; Bruker, 2004 ▶) *T*_min_ = 0.83, *T*_max_ = 0.96
12545 measured reflections
7019 independent reflections
5111 reflections with *I* > 2σ(*I*)
*R*_int_ = 0.029

#### Refinement

*R*[*F* ^2^ > 2σ(*F* ^2^)] = 0.072
*wR*(*F* ^2^) = 0.223
*S* = 1.02
7019 reflections
582 parameters
6 restraints
H-atom parameters constrained
Δρ_max_ = 0.76 e Å^−3^
Δρ_min_ = −0.37 e Å^−3^

### 

Data collection: *SMART* (Bruker, 2004 ▶); cell refinement: *SAINT* (Bruker, 2004 ▶); data reduction: *SAINT*; program(s) used to solve structure: *SHELXS97* (Sheldrick, 2008 ▶); program(s) used to refine structure: *SHELXL97* (Sheldrick, 2008 ▶); molecular graphics: *ORTEP-3 for Windows* (Farrugia, 2012 ▶); software used to prepare material for publication: *SHELXTL* (Sheldrick, 2008 ▶).

## Supplementary Material

Crystal structure: contains datablock(s) I, global. DOI: 10.1107/S1600536813007022/br2221sup1.cif

Structure factors: contains datablock(s) I. DOI: 10.1107/S1600536813007022/br2221Isup2.hkl

Supplementary material file. DOI: 10.1107/S1600536813007022/br2221Isup3.cdx

Additional supplementary materials:  crystallographic information; 3D view; checkCIF report

## Figures and Tables

**Fig. 1.**
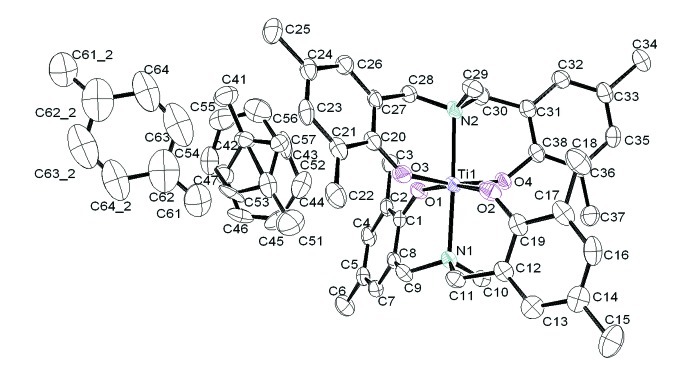
Molecular structure of the title compound, with the atom-numbering scheme. Displacement ellipsoids are drawn at the 50% probability level. H atoms are omitted for clarity. Both positions of the toluene molecule are shown disordered around the center of symmetry.

## supplementary crystallographic information

### Comment

Titanium complexes containing tridentate or tetradentate ligands have attracted considerable attention in the fields of organometallic catalysis. Recently, we have reported the various kinds of titanium complexes containing these ligands (Hong *et al.* 2008, Kim *et al.* 2012, Kim *et al.* 2011, Kim *et al.* 2009, Mun *et al.* 2010, Lee *et al.* 2008, Lee *et al.* 2007). In addition, the similar structure of the title complex with four methylene chloride molecules in the monoclinic unit was reported in the literature (Chmura *et al.*, 2006); however, crystallographic data and parameters for the title compound were quite different from those of the previously reported literature (Chmura *et al.*, 2006). Herein, we report the X-ray structure of the title compound. In the title compound (Fig. 1), Ti atom is coordinated by he two fully deprotonated tridentate ligands *N*-methyl-*N*,*N*-bis(2-oxy-3,5-dimethylbenzyl)amine with 1.5 molecules of toluene in the asymmetric unit. To remove the disorders of toluene molecules, we tried to solve the structure with space group, P1. However, the result was unsuccessful. The coordination geometry around the central Ti atom is close to octahedron.

### Experimental

The title compound could be synthesized in 92% yield (1.18 g) *via* slow addition of Ti(O-^i^Pr)_4_ (0.568 g, 2 mmol) in 10 mL toluene to *N*-methyl-*N*,*N*-bis(2-hydroxy-3,5-dimethylbenzyl)amine (1.20 g, 4 mmol) in 40 mL toluene. The crystal was obtained by slow evaporation of solvent in a refrigerator.

### Refinement

The quality of the crystal used was poor but we were unable to grow a better crystal. We tried refining the structure in the space group P1 to see if this removed the disorder, but this was unsuccessful since it gave non-positive definite atomic displacement parameters. The poor quality of the crystal and the difficulty in clearly resolving the disorder explains the large value of the weighted agreement index.

The disordered toluene molecule was modeled by splitting the atoms into two components (C41—C47 and C51—C57), the site occupation factors of which refined in a ratio of 0.628 (18):0.372 (18). Due to the large thermal parameters, the atomic displacement factor of C57 atom was held fixed. H atoms were positioned geometrically and refined using a riding model, with C—H = 0.93–0.97 Å and with *U*_iso_(H) = 1.2 (1.5 for methyl groups) times *U*_eq_(C). One hydrogen atom in C41 position can not be located in the disordered toluene molecule.

### Crystal data

**Table:**

[Ti(C_19_H_23_NO_2_)_2_]·1.5C_7_H_8_	*Z* = 2
*M**_r_* = 780.34	*F*(000) = 833
Triclinic, *P*1	*D*_x_ = 1.235 Mg m^−^^3^
Hall symbol: -P 1	Mo *K*α radiation, λ = 0.71073 Å
*a* = 12.053 (2) Å	Cell parameters from 7019 reflections
*b* = 13.374 (3) Å	θ = 1.6–24.7°
*c* = 14.511 (3) Å	µ = 0.25 mm^−^^1^
α = 102.02 (3)°	*T* = 173 K
β = 112.07 (3)°	Plate, orange
γ = 93.04 (3)°	0.5 × 0.25 × 0.14 mm
*V* = 2097.8 (7) Å^3^	

### Data collection

**Table:**

Bruker SMART CCD area-detector diffractometer	7019 independent reflections
Radiation source: fine-focus sealed tube	5111 reflections with *I* > 2σ(*I*)
Graphite monochromator	*R*_int_ = 0.029
phi and ω scans	θ_max_ = 24.7°, θ_min_ = 1.6°
Absorption correction: multi-scan (*SADABS*; Bruker, 2004)	*h* = −14→14
*T*_min_ = 0.83, *T*_max_ = 0.96	*k* = −15→15
12545 measured reflections	*l* = −16→17

### Refinement

**Table:**

Refinement on *F*^2^	Primary atom site location: structure-invariant direct methods
Least-squares matrix: full	Secondary atom site location: difference Fourier map
*R*[*F*^2^ > 2σ(*F*^2^)] = 0.072	Hydrogen site location: inferred from neighbouring sites
*wR*(*F*^2^) = 0.223	H-atom parameters constrained
*S* = 1.02	*w* = 1/[σ^2^(*F*_o_^2^) + (0.0838*P*)^2^ + 8.6517*P*] where *P* = (*F*_o_^2^ + 2*F*_c_^2^)/3
7019 reflections	(Δ/σ)_max_ = 0.001
582 parameters	Δρ_max_ = 0.76 e Å^−^^3^
6 restraints	Δρ_min_ = −0.37 e Å^−^^3^

### Special details

**Table:**

Geometry. All e.s.d.'s (except the e.s.d. in the dihedral angle between two l.s. planes) are estimated using the full covariance matrix. The cell e.s.d.'s are taken into account individually in the estimation of e.s.d.'s in distances, angles and torsion angles; correlations between e.s.d.'s in cell parameters are only used when they are defined by crystal symmetry. An approximate (isotropic) treatment of cell e.s.d.'s is used for estimating e.s.d.'s involving l.s. planes.
Refinement. Refinement of *F*^2^ against ALL reflections. The weighted *R*-factor *wR* and goodness of fit *S* are based on *F*^2^, conventional *R*-factors *R* are based on *F*, with *F* set to zero for negative *F*^2^. The threshold expression of *F*^2^ > σ(*F*^2^) is used only for calculating *R*-factors(gt) *etc*. and is not relevant to the choice of reflections for refinement. *R*-factors based on *F*^2^ are statistically about twice as large as those based on *F*, and *R*- factors based on ALL data will be even larger.

### Fractional atomic coordinates and isotropic or equivalent isotropic displacement parameters (Å^2^)

**Table:**

	*x*	*y*	*z*	*U*_iso_*/*U*_eq_	Occ. (<1)
Ti1	0.94677 (8)	0.78673 (6)	0.72763 (7)	0.0252 (2)	
N1	1.0610 (3)	0.9160 (3)	0.7095 (3)	0.0258 (9)	
N2	0.8283 (4)	0.6581 (3)	0.7396 (3)	0.0275 (9)	
O1	0.8214 (3)	0.8631 (3)	0.6767 (3)	0.0317 (8)	
O2	1.0927 (3)	0.7329 (3)	0.7771 (3)	0.0359 (9)	
O3	0.9085 (3)	0.6993 (3)	0.5968 (3)	0.0375 (9)	
O4	0.9677 (3)	0.8483 (2)	0.8632 (2)	0.0312 (8)	
C1	0.7967 (4)	0.9475 (4)	0.6414 (3)	0.0271 (10)	
C2	0.6873 (4)	0.9851 (4)	0.6311 (4)	0.0311 (11)	
C3	0.5956 (5)	0.9261 (5)	0.6540 (5)	0.0438 (14)	
H3A	0.5254	0.9604	0.6418	0.066*	
H3B	0.6298	0.9228	0.7244	0.066*	
H3C	0.5731	0.8574	0.6103	0.066*	
C4	0.6664 (5)	1.0739 (4)	0.5947 (4)	0.0344 (12)	
H4	0.5937	1.0989	0.5866	0.041*	
C5	0.7496 (5)	1.1268 (4)	0.5699 (4)	0.0313 (11)	
C6	0.7247 (5)	1.2233 (4)	0.5318 (5)	0.0443 (14)	
H6A	0.7896	1.2784	0.5744	0.066*	
H6B	0.6502	1.2426	0.5345	0.066*	
H6C	0.7185	1.2105	0.4623	0.066*	
C7	0.8569 (4)	1.0866 (4)	0.5807 (4)	0.0290 (11)	
H7	0.9141	1.1205	0.5648	0.035*	
C8	0.8808 (4)	0.9972 (4)	0.6145 (3)	0.0259 (10)	
C9	0.9900 (4)	0.9480 (4)	0.6142 (4)	0.0297 (11)	
H9A	0.9637	0.8875	0.5568	0.036*	
H9B	1.0432	0.9963	0.6025	0.036*	
C10	1.1006 (4)	1.0068 (4)	0.7986 (4)	0.0296 (11)	
H10A	1.1450	1.0608	0.7865	0.044*	
H10B	1.1514	0.9875	0.8592	0.044*	
H10C	1.0310	1.0310	0.8079	0.044*	
C11	1.1688 (4)	0.8766 (4)	0.6956 (4)	0.0297 (11)	
H11A	1.2114	0.9296	0.6795	0.036*	
H11B	1.1411	0.8170	0.6373	0.036*	
C12	1.2561 (4)	0.8462 (4)	0.7872 (4)	0.0294 (11)	
C13	1.3776 (5)	0.8833 (4)	0.8314 (4)	0.0378 (13)	
H13	1.4072	0.9326	0.8064	0.045*	
C14	1.4576 (5)	0.8495 (4)	0.9121 (4)	0.0394 (13)	
C15	1.5912 (5)	0.8890 (5)	0.9577 (5)	0.0574 (18)	
H15A	1.6294	0.8687	1.0207	0.086*	
H15B	1.6030	0.9630	0.9711	0.086*	
H15C	1.6261	0.8605	0.9103	0.086*	
C16	1.4098 (5)	0.7746 (4)	0.9461 (4)	0.0400 (13)	
H16	1.4620	0.7512	1.0004	0.048*	
C17	1.2885 (5)	0.7331 (4)	0.9035 (4)	0.0369 (13)	
C18	1.2388 (6)	0.6491 (5)	0.9379 (5)	0.0499 (16)	
H18A	1.3043	0.6242	0.9852	0.075*	
H18B	1.1929	0.5933	0.8794	0.075*	
H18C	1.1873	0.6760	0.9710	0.075*	
C19	1.2112 (5)	0.7705 (4)	0.8222 (4)	0.0310 (11)	
C20	0.8330 (5)	0.6163 (4)	0.5304 (4)	0.0301 (11)	
C21	0.8512 (5)	0.5705 (4)	0.4415 (4)	0.0354 (12)	
C22	0.9541 (6)	0.6143 (5)	0.4224 (4)	0.0448 (14)	
H22A	0.9527	0.5749	0.3585	0.067*	
H22B	1.0288	0.6115	0.4771	0.067*	
H22C	0.9472	0.6848	0.4193	0.067*	
C23	0.7690 (5)	0.4852 (4)	0.3731 (4)	0.0357 (12)	
H23	0.7794	0.4550	0.3137	0.043*	
C24	0.6713 (5)	0.4426 (4)	0.3895 (4)	0.0348 (12)	
C25	0.5840 (5)	0.3506 (4)	0.3124 (4)	0.0454 (14)	
H25A	0.5438	0.3162	0.3456	0.068*	
H25B	0.6275	0.3037	0.2845	0.068*	
H25C	0.5251	0.3735	0.2581	0.068*	
C26	0.6599 (5)	0.4878 (4)	0.4799 (4)	0.0337 (12)	
H26	0.5985	0.4592	0.4948	0.040*	
C27	0.7373 (5)	0.5742 (4)	0.5487 (4)	0.0299 (11)	
C28	0.7169 (5)	0.6265 (4)	0.6435 (4)	0.0314 (11)	
H28A	0.6792	0.6874	0.6309	0.038*	
H28B	0.6607	0.5798	0.6536	0.038*	
C29	0.8925 (5)	0.5682 (4)	0.7575 (4)	0.0352 (12)	
H29A	0.8380	0.5128	0.7570	0.053*	
H29B	0.9598	0.5875	0.8229	0.053*	
H29C	0.9212	0.5459	0.7043	0.053*	
C30	0.7875 (4)	0.6935 (4)	0.8250 (4)	0.0306 (11)	
H30A	0.7419	0.7503	0.8116	0.037*	
H30B	0.7334	0.6375	0.8253	0.037*	
C31	0.8885 (4)	0.7281 (4)	0.9288 (4)	0.0293 (11)	
C32	0.8961 (5)	0.6846 (4)	1.0083 (4)	0.0348 (12)	
H32	0.8392	0.6288	0.9972	0.042*	
C33	0.9879 (5)	0.7228 (4)	1.1055 (4)	0.0361 (12)	
C34	0.9934 (6)	0.6776 (5)	1.1945 (4)	0.0502 (15)	
H34A	1.0762	0.6741	1.2355	0.075*	
H34B	0.9480	0.6094	1.1685	0.075*	
H34C	0.9596	0.7207	1.2357	0.075*	
C35	1.0725 (5)	0.8038 (4)	1.1196 (4)	0.0320 (11)	
H35	1.1335	0.8296	1.1846	0.038*	
C36	1.0708 (5)	0.8492 (4)	1.0406 (4)	0.0295 (11)	
C37	1.1644 (5)	0.9370 (4)	1.0572 (4)	0.0414 (13)	
H37A	1.2115	0.9147	1.0182	0.062*	
H37B	1.2164	0.9589	1.1287	0.062*	
H37C	1.1251	0.9935	1.0352	0.062*	
C38	0.9765 (5)	0.8091 (4)	0.9438 (4)	0.0284 (11)	
C41	0.3393 (13)	0.6314 (13)	0.2349 (12)	0.060 (4)	0.628 (18)
H41A	0.2724	0.6627	0.2425	0.090*	0.628 (18)
H41B	0.3573	0.5800	0.2737	0.090*	0.628 (18)
H41C	0.3186	0.5995	0.1638	0.090*	0.628 (18)
C42	0.4496 (11)	0.7138 (8)	0.2742 (8)	0.038 (3)	0.628 (18)
C43	0.558 (4)	0.712 (3)	0.363 (3)	0.056 (10)	0.628 (18)
H43	0.5629	0.6581	0.3953	0.068*	0.628 (18)
C44	0.6493 (12)	0.7890 (9)	0.3965 (11)	0.060 (4)	0.628 (18)
H44	0.7160	0.7917	0.4566	0.072*	0.628 (18)
C45	0.6473 (16)	0.8630 (10)	0.3456 (13)	0.077 (5)	0.628 (18)
H45	0.7131	0.9151	0.3716	0.093*	0.628 (18)
C46	0.5523 (19)	0.8639 (13)	0.2574 (14)	0.084 (7)	0.628 (18)
H46	0.5551	0.9119	0.2200	0.101*	0.628 (18)
C47	0.450 (2)	0.7896 (14)	0.2252 (16)	0.044 (5)	0.628 (18)
H47	0.3811	0.7924	0.1690	0.052*	0.628 (18)
C51	0.720 (3)	0.829 (2)	0.362 (2)	0.075 (7)	0.372 (18)
H51A	0.7702	0.7923	0.4075	0.113*	0.372 (18)
H51B	0.7252	0.8991	0.3985	0.113*	0.372 (18)
H51C	0.7463	0.8294	0.3070	0.113*	0.372 (18)
C52	0.5938 (17)	0.7787 (14)	0.3187 (14)	0.047 (6)	0.372 (18)
C53	0.510 (3)	0.813 (3)	0.238 (3)	0.049 (10)	0.372 (18)
H53	0.5337	0.8691	0.2167	0.058*	0.372 (18)
C54	0.395 (3)	0.764 (3)	0.192 (2)	0.052 (9)	0.372 (18)
H54	0.3406	0.7830	0.1354	0.062*	0.372 (18)
C55	0.356 (3)	0.6849 (19)	0.226 (2)	0.056 (6)	0.372 (18)
H55	0.2762	0.6531	0.1945	0.067*	0.372 (18)
C56	0.437 (2)	0.6543 (19)	0.3067 (18)	0.064 (7)	0.372 (18)
H56	0.4144	0.6004	0.3305	0.076*	0.372 (18)
C57	0.547 (5)	0.704 (4)	0.349 (4)	0.035 (12)	0.372 (18)
H57	0.5999	0.6863	0.4069	0.042*	0.372 (18)
C61	0.6733 (13)	0.6495 (13)	0.1397 (12)	0.071 (4)	0.50
H61A	0.6362	0.6937	0.1773	0.106*	0.50
H61B	0.7118	0.6897	0.1095	0.106*	0.50
H61C	0.7325	0.6180	0.1855	0.106*	0.50
C62	0.5915 (9)	0.5786 (8)	0.0683 (8)	0.092 (3)	
C63	0.5316 (11)	0.5130 (9)	0.1048 (8)	0.095 (3)	
H63	0.5534	0.5217	0.1749	0.114*	
C64	0.4435 (9)	0.4383 (8)	0.0378 (7)	0.083 (3)	
H64	0.4039	0.3958	0.0626	0.099*	

### Atomic displacement parameters (Å^2^)

**Table:**

	*U*^11^	*U*^22^	*U*^33^	*U*^12^	*U*^13^	*U*^23^
Ti1	0.0311 (5)	0.0249 (4)	0.0234 (5)	0.0041 (4)	0.0141 (4)	0.0079 (3)
N1	0.028 (2)	0.030 (2)	0.024 (2)	0.0085 (17)	0.0123 (18)	0.0095 (17)
N2	0.036 (2)	0.030 (2)	0.020 (2)	0.0053 (18)	0.0147 (18)	0.0073 (17)
O1	0.0305 (19)	0.0367 (19)	0.034 (2)	0.0059 (15)	0.0142 (16)	0.0171 (16)
O2	0.041 (2)	0.0318 (19)	0.045 (2)	0.0120 (16)	0.0227 (19)	0.0181 (17)
O3	0.049 (2)	0.037 (2)	0.029 (2)	−0.0059 (17)	0.0225 (18)	0.0030 (16)
O4	0.046 (2)	0.0262 (17)	0.0214 (18)	0.0005 (15)	0.0134 (16)	0.0060 (14)
C1	0.028 (3)	0.031 (3)	0.017 (2)	0.002 (2)	0.005 (2)	0.004 (2)
C2	0.028 (3)	0.044 (3)	0.019 (2)	0.004 (2)	0.006 (2)	0.009 (2)
C3	0.026 (3)	0.067 (4)	0.043 (3)	0.009 (3)	0.010 (3)	0.028 (3)
C4	0.027 (3)	0.049 (3)	0.029 (3)	0.015 (2)	0.008 (2)	0.016 (2)
C5	0.033 (3)	0.035 (3)	0.023 (3)	0.010 (2)	0.005 (2)	0.010 (2)
C6	0.046 (3)	0.049 (3)	0.048 (4)	0.022 (3)	0.021 (3)	0.026 (3)
C7	0.032 (3)	0.032 (3)	0.023 (3)	0.004 (2)	0.011 (2)	0.007 (2)
C8	0.028 (3)	0.031 (3)	0.019 (2)	0.004 (2)	0.010 (2)	0.007 (2)
C9	0.034 (3)	0.035 (3)	0.028 (3)	0.009 (2)	0.017 (2)	0.015 (2)
C10	0.030 (3)	0.029 (3)	0.031 (3)	0.002 (2)	0.013 (2)	0.009 (2)
C11	0.030 (3)	0.033 (3)	0.034 (3)	0.008 (2)	0.018 (2)	0.012 (2)
C12	0.032 (3)	0.033 (3)	0.030 (3)	0.013 (2)	0.018 (2)	0.009 (2)
C13	0.041 (3)	0.033 (3)	0.042 (3)	0.012 (2)	0.019 (3)	0.006 (2)
C14	0.038 (3)	0.038 (3)	0.039 (3)	0.016 (2)	0.016 (3)	0.002 (3)
C15	0.042 (4)	0.060 (4)	0.053 (4)	0.015 (3)	0.007 (3)	−0.001 (3)
C16	0.040 (3)	0.051 (3)	0.028 (3)	0.026 (3)	0.011 (2)	0.008 (2)
C17	0.049 (3)	0.038 (3)	0.037 (3)	0.024 (3)	0.027 (3)	0.015 (2)
C18	0.054 (4)	0.059 (4)	0.057 (4)	0.029 (3)	0.032 (3)	0.033 (3)
C19	0.036 (3)	0.034 (3)	0.028 (3)	0.015 (2)	0.017 (2)	0.009 (2)
C20	0.041 (3)	0.026 (2)	0.025 (3)	0.009 (2)	0.015 (2)	0.008 (2)
C21	0.051 (3)	0.034 (3)	0.027 (3)	0.015 (2)	0.018 (3)	0.012 (2)
C22	0.060 (4)	0.051 (3)	0.032 (3)	0.013 (3)	0.027 (3)	0.009 (3)
C23	0.049 (3)	0.038 (3)	0.019 (3)	0.018 (3)	0.012 (2)	0.004 (2)
C24	0.050 (3)	0.027 (3)	0.025 (3)	0.012 (2)	0.011 (2)	0.006 (2)
C25	0.052 (4)	0.044 (3)	0.032 (3)	0.007 (3)	0.012 (3)	0.000 (3)
C26	0.042 (3)	0.028 (3)	0.029 (3)	0.005 (2)	0.013 (2)	0.005 (2)
C27	0.043 (3)	0.030 (3)	0.018 (2)	0.006 (2)	0.013 (2)	0.006 (2)
C28	0.039 (3)	0.033 (3)	0.023 (3)	−0.002 (2)	0.015 (2)	0.005 (2)
C29	0.047 (3)	0.029 (3)	0.037 (3)	0.006 (2)	0.023 (3)	0.010 (2)
C30	0.031 (3)	0.038 (3)	0.024 (3)	0.002 (2)	0.016 (2)	0.003 (2)
C31	0.031 (3)	0.035 (3)	0.027 (3)	0.008 (2)	0.016 (2)	0.009 (2)
C32	0.041 (3)	0.038 (3)	0.029 (3)	0.001 (2)	0.018 (2)	0.008 (2)
C33	0.040 (3)	0.046 (3)	0.026 (3)	0.010 (3)	0.015 (2)	0.014 (2)
C34	0.061 (4)	0.057 (4)	0.033 (3)	0.004 (3)	0.013 (3)	0.023 (3)
C35	0.032 (3)	0.039 (3)	0.023 (3)	0.011 (2)	0.009 (2)	0.005 (2)
C36	0.035 (3)	0.031 (3)	0.027 (3)	0.009 (2)	0.017 (2)	0.005 (2)
C37	0.047 (3)	0.045 (3)	0.027 (3)	−0.004 (3)	0.014 (3)	0.001 (2)
C38	0.036 (3)	0.031 (3)	0.023 (3)	0.011 (2)	0.016 (2)	0.007 (2)
C41	0.051 (8)	0.061 (9)	0.061 (9)	−0.013 (8)	0.024 (7)	0.005 (8)
C42	0.046 (8)	0.035 (6)	0.041 (6)	0.001 (5)	0.025 (6)	0.009 (5)
C43	0.085 (18)	0.055 (16)	0.021 (10)	0.021 (12)	0.006 (10)	0.016 (9)
C44	0.056 (8)	0.059 (8)	0.043 (8)	0.003 (6)	0.004 (6)	−0.003 (6)
C45	0.077 (11)	0.036 (8)	0.095 (12)	−0.023 (7)	0.024 (10)	−0.009 (7)
C46	0.117 (17)	0.066 (10)	0.071 (11)	−0.036 (9)	0.036 (11)	0.033 (9)
C47	0.060 (17)	0.035 (9)	0.034 (9)	0.006 (12)	0.014 (11)	0.014 (7)
C51	0.080 (18)	0.062 (15)	0.077 (16)	0.001 (13)	0.030 (15)	0.010 (12)
C52	0.057 (12)	0.054 (12)	0.032 (12)	0.020 (9)	0.018 (10)	0.012 (8)
C53	0.05 (2)	0.07 (2)	0.06 (2)	0.02 (2)	0.04 (2)	0.05 (2)
C54	0.05 (2)	0.07 (2)	0.034 (15)	0.025 (16)	0.011 (12)	0.007 (12)
C55	0.058 (17)	0.043 (14)	0.070 (15)	0.006 (14)	0.036 (14)	0.002 (13)
C56	0.071 (18)	0.063 (14)	0.077 (17)	0.019 (13)	0.047 (15)	0.022 (13)
C57	0.038 (14)	0.033 (14)	0.033 (15)	0.000 (8)	0.013 (10)	0.006 (8)
C61	0.049 (8)	0.099 (12)	0.065 (10)	0.029 (9)	0.020 (8)	0.021 (9)
C62	0.094 (7)	0.103 (7)	0.084 (7)	0.074 (6)	0.033 (6)	0.020 (6)
C63	0.137 (9)	0.111 (8)	0.074 (6)	0.093 (7)	0.061 (7)	0.043 (6)
C64	0.104 (7)	0.106 (7)	0.061 (5)	0.072 (6)	0.042 (5)	0.037 (5)

### Geometric parameters (Å, º)

**Table:**

Ti1—O1	1.873 (3)	C25—H25B	0.9600
Ti1—O2	1.879 (4)	C25—H25C	0.9600
Ti1—O4	1.882 (3)	C26—C27	1.381 (7)
Ti1—O3	1.882 (4)	C26—H26	0.9300
Ti1—N2	2.254 (4)	C27—C28	1.521 (6)
Ti1—N1	2.272 (4)	C28—H28A	0.9700
N1—C10	1.482 (6)	C28—H28B	0.9700
N1—C9	1.491 (6)	C29—H29A	0.9600
N1—C11	1.494 (6)	C29—H29B	0.9600
N2—C29	1.479 (6)	C29—H29C	0.9600
N2—C28	1.488 (6)	C30—C31	1.496 (7)
N2—C30	1.496 (6)	C30—H30A	0.9700
O1—C1	1.337 (6)	C30—H30B	0.9700
O2—C19	1.343 (6)	C31—C32	1.372 (7)
O3—C20	1.334 (6)	C31—C38	1.398 (7)
O4—C38	1.349 (6)	C32—C33	1.394 (7)
C1—C8	1.398 (7)	C32—H32	0.9300
C1—C2	1.403 (7)	C33—C35	1.375 (8)
C2—C4	1.394 (7)	C33—C34	1.516 (7)
C2—C3	1.498 (7)	C34—H34A	0.9600
C3—H3A	0.9600	C34—H34B	0.9600
C3—H3B	0.9600	C34—H34C	0.9600
C3—H3C	0.9600	C35—C36	1.399 (7)
C4—C5	1.392 (7)	C35—H35	0.9300
C4—H4	0.9300	C36—C38	1.405 (7)
C5—C7	1.394 (7)	C36—C37	1.499 (7)
C5—C6	1.509 (7)	C37—H37A	0.9600
C6—H6A	0.9600	C37—H37B	0.9600
C6—H6B	0.9600	C37—H37C	0.9600
C6—H6C	0.9600	C41—C42	1.523 (18)
C7—C8	1.389 (7)	C41—H41A	0.9600
C7—H7	0.9300	C41—H41B	0.9600
C8—C9	1.504 (6)	C41—H41C	0.9600
C9—H9A	0.9700	C42—C47	1.35 (2)
C9—H9B	0.9700	C42—C43	1.45 (4)
C10—H10A	0.9600	C43—C44	1.34 (4)
C10—H10B	0.9600	C43—H43	0.9300
C10—H10C	0.9600	C44—C45	1.35 (2)
C11—C12	1.502 (7)	C44—H44	0.9300
C11—H11A	0.9700	C45—C46	1.36 (2)
C11—H11B	0.9700	C45—H45	0.9300
C12—C13	1.374 (7)	C46—C47	1.40 (2)
C12—C19	1.393 (7)	C46—H46	0.9300
C13—C14	1.387 (8)	C47—H47	0.9300
C13—H13	0.9300	C51—C52	1.47 (3)
C14—C16	1.392 (8)	C51—H51A	0.9600
C14—C15	1.508 (8)	C51—H51B	0.9600
C15—H15A	0.9600	C51—H51C	0.9600
C15—H15B	0.9600	C52—C57	1.34 (5)
C15—H15C	0.9600	C52—C53	1.41 (3)
C16—C17	1.385 (8)	C53—C54	1.36 (4)
C16—H16	0.9300	C53—H53	0.9300
C17—C19	1.407 (7)	C54—C55	1.39 (5)
C17—C18	1.503 (8)	C54—H54	0.9300
C18—H18A	0.9600	C55—C56	1.37 (4)
C18—H18B	0.9600	C55—H55	0.9300
C18—H18C	0.9600	C56—C57	1.31 (6)
C20—C27	1.393 (7)	C56—H56	0.9300
C20—C21	1.409 (7)	C57—H57	0.9300
C21—C23	1.392 (8)	C61—C62	1.292 (17)
C21—C22	1.485 (8)	C61—H61A	0.9600
C22—H22A	0.9600	C61—H61B	0.9600
C22—H22B	0.9600	C61—H61C	0.9600
C22—H22C	0.9600	C62—C64^i^	1.399 (12)
C23—C24	1.401 (8)	C62—C63	1.408 (14)
C23—H23	0.9300	C63—C64	1.334 (13)
C24—C26	1.386 (7)	C63—H63	0.9300
C24—C25	1.509 (8)	C64—C62^i^	1.399 (12)
C25—H25A	0.9600	C64—H64	0.9300
			
O1—Ti1—O2	168.10 (15)	C27—C26—H26	119.1
O1—Ti1—O4	90.78 (15)	C24—C26—H26	119.1
O2—Ti1—O4	90.01 (16)	C26—C27—C20	120.3 (5)
O1—Ti1—O3	92.34 (17)	C26—C27—C28	120.5 (5)
O2—Ti1—O3	89.53 (17)	C20—C27—C28	119.2 (4)
O4—Ti1—O3	167.03 (15)	N2—C28—C27	114.9 (4)
O1—Ti1—N2	95.28 (15)	N2—C28—H28A	108.6
O2—Ti1—N2	96.62 (15)	C27—C28—H28A	108.6
O4—Ti1—N2	85.06 (15)	N2—C28—H28B	108.6
O3—Ti1—N2	82.12 (15)	C27—C28—H28B	108.6
O1—Ti1—N1	83.29 (14)	H28A—C28—H28B	107.5
O2—Ti1—N1	84.82 (15)	C31—C30—N2	114.1 (4)
O4—Ti1—N1	96.52 (15)	C31—C30—H30A	108.7
O3—Ti1—N1	96.34 (15)	N2—C30—H30A	108.7
N2—Ti1—N1	177.87 (15)	C31—C30—H30B	108.7
C10—N1—C9	109.4 (4)	N2—C30—H30B	108.7
C10—N1—C11	109.9 (4)	H30A—C30—H30B	107.6
C9—N1—C11	106.6 (3)	C32—C31—C38	119.9 (5)
C10—N1—Ti1	112.1 (3)	C32—C31—C30	122.7 (5)
C9—N1—Ti1	109.4 (3)	C38—C31—C30	117.4 (4)
C11—N1—Ti1	109.4 (3)	C31—C32—C33	120.9 (5)
C29—N2—C28	110.0 (4)	C31—C32—H32	119.5
C29—N2—C30	108.6 (4)	C33—C32—H32	119.5
C28—N2—C30	106.5 (4)	C35—C33—C32	118.5 (5)
C29—N2—Ti1	111.4 (3)	C35—C33—C34	120.5 (5)
C28—N2—Ti1	109.1 (3)	C32—C33—C34	121.0 (5)
C30—N2—Ti1	111.1 (3)	C33—C35—C36	122.9 (5)
C1—O1—Ti1	142.9 (3)	C33—C35—H35	118.6
C19—O2—Ti1	136.5 (3)	C36—C35—H35	118.6
C20—O3—Ti1	141.8 (3)	C35—C36—C38	117.0 (5)
C38—O4—Ti1	132.7 (3)	C35—C36—C37	122.2 (5)
O1—C1—C8	119.2 (4)	C38—C36—C37	120.8 (4)
O1—C1—C2	120.2 (4)	O4—C38—C31	118.3 (4)
C8—C1—C2	120.6 (4)	O4—C38—C36	121.1 (4)
C4—C2—C1	117.9 (5)	C31—C38—C36	120.7 (4)
C4—C2—C3	122.8 (5)	C47—C42—C43	118.3 (19)
C1—C2—C3	119.3 (5)	C47—C42—C41	120.4 (15)
C5—C4—C2	123.0 (5)	C43—C42—C41	121.2 (19)
C5—C4—H4	118.5	C44—C43—C42	118 (3)
C2—C4—H4	118.5	C44—C43—H43	120.8
C4—C5—C7	117.4 (4)	C42—C43—H43	120.8
C4—C5—C6	121.6 (5)	C43—C44—C45	121.7 (19)
C7—C5—C6	121.0 (5)	C43—C44—H44	119.2
C8—C7—C5	121.8 (5)	C45—C44—H44	119.2
C8—C7—H7	119.1	C44—C45—C46	122.3 (15)
C5—C7—H7	119.1	C44—C45—H45	118.9
C7—C8—C1	119.3 (4)	C46—C45—H45	118.9
C7—C8—C9	121.1 (4)	C45—C46—C47	117.4 (17)
C1—C8—C9	119.3 (4)	C45—C46—H46	121.3
N1—C9—C8	115.6 (4)	C47—C46—H46	121.3
N1—C9—H9A	108.4	C42—C47—C46	121.5 (18)
C8—C9—H9A	108.4	C42—C47—H47	119.2
N1—C9—H9B	108.4	C46—C47—H47	119.2
C8—C9—H9B	108.4	C52—C51—H51A	109.5
H9A—C9—H9B	107.4	C52—C51—H51B	109.5
N1—C11—C12	114.4 (4)	H51A—C51—H51B	109.5
N1—C11—H11A	108.7	C52—C51—H51C	109.5
C12—C11—H11A	108.7	H51A—C51—H51C	109.5
N1—C11—H11B	108.7	H51B—C51—H51C	109.5
C12—C11—H11B	108.7	C57—C52—C53	116 (3)
H11A—C11—H11B	107.6	C57—C52—C51	127 (3)
C13—C12—C19	119.5 (5)	C53—C52—C51	117 (2)
C13—C12—C11	123.1 (5)	C54—C53—C52	118 (3)
C19—C12—C11	117.1 (4)	C54—C53—H53	120.8
C12—C13—C14	122.0 (5)	C52—C53—H53	120.8
C12—C13—H13	119.0	C53—C54—C55	122 (3)
C14—C13—H13	119.0	C53—C54—H54	119.1
C13—C14—C16	117.1 (5)	C55—C54—H54	119.1
C13—C14—C15	121.2 (6)	C56—C55—C54	119 (3)
C16—C14—C15	121.7 (5)	C56—C55—H55	120.3
C17—C16—C14	123.5 (5)	C54—C55—H55	120.3
C17—C16—H16	118.3	C57—C56—C55	117 (4)
C14—C16—H16	118.3	C57—C56—H56	121.5
C16—C17—C19	117.2 (5)	C55—C56—H56	121.5
C16—C17—C18	123.0 (5)	C56—C57—C52	128 (5)
C19—C17—C18	119.8 (5)	C56—C57—H57	116.2
O2—C19—C12	119.2 (4)	C52—C57—H57	116.2
O2—C19—C17	120.1 (5)	C62—C61—H61A	109.5
C12—C19—C17	120.7 (5)	C62—C61—H61B	109.5
O3—C20—C27	120.6 (4)	H61A—C61—H61B	109.5
O3—C20—C21	119.5 (5)	C62—C61—H61C	109.5
C27—C20—C21	120.0 (5)	H61A—C61—H61C	109.5
C23—C21—C20	117.7 (5)	H61B—C61—H61C	109.5
C23—C21—C22	122.2 (5)	C61—C62—C64^i^	127.7 (14)
C20—C21—C22	120.1 (5)	C61—C62—C63	114.1 (12)
C21—C23—C24	123.2 (5)	C64^i^—C62—C63	118.2 (10)
C21—C23—H23	118.4	C64—C63—C62	119.4 (9)
C24—C23—H23	118.4	C64—C63—H63	120.3
C26—C24—C23	117.0 (5)	C62—C63—H63	120.3
C26—C24—C25	121.9 (5)	C63—C64—C62^i^	122.4 (10)
C23—C24—C25	121.1 (5)	C63—C64—H64	118.8
C27—C26—C24	121.8 (5)	C62^i^—C64—H64	118.8

Symmetry code: (i) −*x*+1, −*y*+1, −*z*.
